# Nursing Students' Knowledge and Attitudes Toward Alzheimer's Disease in Saudi Arabia: A Cross-Sectional Study

**DOI:** 10.7759/cureus.61444

**Published:** 2024-05-31

**Authors:** Jazi S Alotaibi

**Affiliations:** 1 Department of Nursing, College of Applied Medical Sciences, Majmaah University, Majmaah, SAU

**Keywords:** saudi arabia, bachelor’s nursing students, attitudes, knowledge, alzheimer's disease

## Abstract

Background

The elderly population continues to grow worldwide, including in Saudi Arabia. Caring for older people with Alzheimer's and dementia disease is very challenging and merits specific skills, knowledge, and attitudes among nurses and nursing students. Consequently, nursing students must be prepared with the appropriate knowledge and attitude to care for patients affected by Alzheimer’s in their future professions. This study aimed to investigate the knowledge and attitudes of Alzheimer’s disease (AD) among bachelor’s nursing students in Saudi Arabia.

Methods

This study used a descriptive cross-sectional design, and data were collected via an online questionnaire comprising two main instruments: the Alzheimer’s Disease Knowledge Scale (ADKS) and the Dementia Attitudes Scale (DAS). A total of 477 undergraduate nursing students participated in the study at four universities in four regions of Saudi Arabia.

Result

The results indicated that Saudi nursing students exhibited insufficient knowledge regarding individuals with AD, reflected by a mean ADKS score of 13.83 out of 30. Yet, they displayed positive attitudes, as indicated by a mean DAS score of 99.29 out of 140. Nursing students in their third year and those who had family members with AD had a higher significant score regarding their knowledge of AD than nursing students who were in their fourth year or those who did not have family members who had AD. Additionally, nursing students aged 20 to 25 years, as well as nursing students in their fourth year, had more positive attitudes toward working with AD patients.

Conclusions

In conclusion, this study revealed that although many nursing students have a positive attitude toward working with AD patients, they have insufficient knowledge of AD. Therefore, there is an urgent necessity for enhanced educational initiatives, encompassing both greater depth and improved quality, as well as increased clinical training to address this knowledge gap among nursing students in Saudi Arabia.

## Introduction

The size of the population of older individuals is expected to increase in the future. The World Health Organization [1] states that the number of individuals aged over 60 years worldwide is expected to rise substantially, from 1 billion in 2020 to 2.1 billion by 2050. This demographic shift would mean that approximately 22% of the global population at that time would comprise individuals aged 60 years and older. Furthermore, as people age, they are likely to experience health problems, including dementia [2]. Currently, dementia afflicts over 50 million individuals; this number is expected to triple by 2050 and, accordingly, increase healthcare costs [1]. This illness most commonly manifests as Alzheimer’s disease (AD), which may involve 60%−70% of dementia cases [3]. The Saudi Arabian Ministry of Health [4] reports that in Saudi Arabia, about 130,000 patients were diagnosed with AD in 2020. This number is anticipated to rise significantly due to the aging population in Saudi Arabia, with people over 60 years expected to increase from 4.4% of the total population in 2021 to around 18% by 2050 [5]. Consequently, there will be a growing demand for professional nurses in caring for AD patients to meet the increasing needs of this population.

AD is a type of dementia that is accompanied by symptoms that are expected to worsen over time, including confusion, poor judgment, and difficulty communicating and remembering [2]. In 2019, official death records reported approximately 121,499 deaths attributed to AD, which position it as the sixth-greatest cause of death in the United States [2]. The cost of care for AD and dementia-related expenditures in the United States amounts to more than US$1.3 trillion annually and is expected to be approximately US$2.8 trillion annually by 2030. Half of this cost is related to informal care [6].

This situation has caused considerable demand for nurses who work with people with AD. However, a significant proportion of nurses have no fundamental training in geriatrics or in providing care for older adults [7], which indicates a severe shortage of registered nurses who can work in elderly care. Nurses play essential roles in caring for AD patients who have impaired mental functioning; however, nurses find these roles overwhelming [8]. Another challenge is that aspiring nurses lack the knowledge required to provide appropriate care to AD patients [9]. The failure of nurses to understand the unmet needs of AD patients may lead to inappropriate and ineffective care. The knowledge and attitudes of nursing students regarding AD are pivotal to their delivery of effective patient care, ensuring patient safety, and reducing stigma. If nurses have an understanding of the disease, it would also enhance the quality of life of patients and enable nursing students, as future professional nurses, to advocate for improved support. Therefore, an evaluation of this knowledge should be a requirement to enhance education about this disease by nursing curricula [10].

Many studies have explored the knowledge of nursing students about AD and the literature demonstrates that this knowledge differs across countries and cultural backgrounds. Some studies found that nursing students have an average or high level of knowledge of AD [11-14]. However, most studies report low levels of knowledge of AD [15-19]. Students who lack knowledge of AD are likely to encounter challenges in their care for patients with AD [20]. Furthermore, deficits in the knowledge, skills, and attitudes of staff and students regarding AD cause them to struggle to provide care, particularly in relation to nutrition, mobility, emotional requirements, and communication professions [20]. These issues highlight the need for nursing institutions to modify their curricula by incorporating specialized courses and training that are intended to improve the quality of care provided for people with AD [20].

Having knowledge alone is insufficient when it comes to delivering optimal care to individuals with AD. It is equally important for nursing students to possess positive attitudes toward patients. Such attitudes have been assessed by many studies and reported positive attitudes among nursing students in different settings [11,12,17,19,21,22]. However, other studies uncovered negative attitudes toward AD among nursing students [23]. Possessing positive attitudes toward patients with AD among nursing students can positively impact the students' clinical experience with the patients [13].

In addition, it has been found that age relates positively [11,14] or negatively [24] to the attitudes of nursing students regarding AD. Nursing students at a later stage of their education tend to exhibit higher levels of knowledge and more positive attitudes toward AD than students at earlier stages of their studies [11-13]. However, other studies have shown no statistical difference in attitudes or knowledge between students of different academic levels [17].

Even though numerous studies in different countries have delved into student nurses’ knowledge and attitudes toward patients with AD, no studies were found on this topic in the context of Saudi Arabia. To address this deficiency, the present research enquired about the knowledge of and attitudes toward people living with AD of nursing students in Saudi Arabia and explored the factors that affect these constructs. The investigation pursued the following objectives: (1) To assess the knowledge of Saudi nursing students about AD; (2) to evaluate their attitudes toward the disease; (3) to determine the relationship between the sociodemographic characteristics of the students and their knowledge of AD; and (4) to determine the relationship between the sociodemographic characteristics of the students and their attitudes toward AD.

Considering that it is expected that the number of older adults and AD patients will increase in Saudi Arabia in the future, it is essential to investigate the knowledge and attitudes of nursing students toward working with patients with AD. This investigation could guide nursing schools in developing the curriculum to ensure comprehensive education in geriatric and dementia care, including AD care, to improve comprehensive training in geriatric and dementia care while cultivating favorable attitudes in nursing students about caring for AD patients and promoting excellence and empathy in healthcare provision in the Saudi Arabian context.

## Materials and methods

Method

The study used a cross-sectional design. Such research design enables the researcher to assess participants' exposure to the studied phenomenon.

Participants and settings

This study was performed at four universities in different regions of Saudi Arabia: Majmaah University (central region), Al-Jouf University (northern region), Hafer Al-Batin University (eastern region) and Taif University (western region). Because of difficulties in communication and time constraints, no university in the southern region of Saudi Arabia was included in the study. The study was conducted at either an independent nursing college, such as the College of Nursing at Jouf University, or at colleges of applied medical sciences that contained nursing departments as one of their departments, such as the other three universities involved.

Convenience sampling was employed in this study and the target population was all nursing students who met the inclusion criteria at the selected universities. The inclusion criterion was nursing students only in their third and fourth years of a nursing program. The rationale behind targeting third and fourth-year students was that it was anticipated that they had been exposed to geriatrics courses and had gained knowledge of caring for elderly patients.

Currently, approximately 19,000 students are studying in government nursing schools in Saudi Arabia. The required sample size was determined using G* Power Version 3.1 (Heinrich Heine University Düsseldorf, Germany) and considering an effect size of 0.25 and an alpha error of 0.05. The objective of this analysis was to establish the smallest number of participants required to identify the effect of a specific test at the desired level of significance. The power analysis suggested a minimum sample size of 377 for implementing an ANOVA test. The questionnaires were distributed to all nursing students at the four selected colleges who met the eligibility criterion - about 806 students in total. Of these, 477 nursing students completed and returned the questionnaire, which gave a 59.1% response rate.

Data collection

Data collection was performed from August 22, 2023, to October 22, 2023. First, the researcher visited the research center at each university as the first contact to obtain a referral to the college chosen for the study. Subsequently, the researcher met the deans and then, the heads of the department and provided them with information about the study. Then, questionnaires were distributed to the nursing students via their university email to ensure effective and secure delivery. The questionnaire included an introductory letter inviting them to participate, containing essential information, such as the study's aim, objectives, and significance. It also emphasized the voluntary nature of involvement. Students were asked to respond to the survey questions using the provided Survey Monkey link. It was estimated that it would take approximately 25 to 35 minutes to complete the survey. To increase the response rate, the researcher sent a reminder email every two weeks to remind them to participate in this study.

Ethical considerations

First, this study was approved by the Institutional Review Board of Majmaah University (approval no. MUREC-June. I 9/COM-2023/23-11). To protect subjects’ privacy, all data were treated with strict confidentiality. The online survey asked no questions that could identify the participants which ensured their anonymity; therefore, no personal information was collected [25]. The participants were informed that their involvement in the study was entirely voluntary and that they could withdraw at any time [25]. All electronic data were securely stored in password-protected files and access to these data was limited to the research team. There was no risk to the participants in this study and even though no benefits would accrue to the respondents [25], it was anticipated that the study outcomes would lead to recommendations for nursing education and practice in Saudi Arabia in the future. All data will be kept secure and used for this study only. The data will be destroyed three years after the study or as directed by the International Review Board. Before they started completing the electronic questionaries, the students recorded consent by selecting ‎the yes option in response to an item that specifically asked them to consent to participation.

Instruments of the study

Two established instruments were used in this study. The first instrument, the AD Knowledge Scale (ADKS), developed by Carpenter et al. [26], comprises 30 items that require yes/no responses. To calculate the score, correct answers were scored 1 and wrong answers 0, which led to a total score between 0 and 30. This instrument is to be used with many participants, including health and nursing students, healthcare professionals, and nurses. The questions cover seven key areas of AD knowledge, that is, life impact, symptoms, risk factors, treatment and management, assessment and diagnosis, caregiving, and course of the disease. Carpenter et al. [26] analyzed the psychometric attributes of the scale and found that it demonstrates adequate reliability and validity in terms of content and predictive, concurrent, and convergent measures. Because of its high reliability and validity, as demonstrated by other studies [11,22,27,28] the ADKS was chosen for this study to evaluate nursing students’ knowledge regarding AD.

The second instrument measured the respondents’ attitudes toward dementia. The Dementia Attitudes Scale (DAS) [29] is a 20-item scale that measures the emotional, behavioral, and cognitive dimensions of attitudes toward AD and other dementias. A seven-point Likert scale ranging from 1 (strongly disagree) to 7 (strongly agree) records responses to the items. It comprises two main factors namely dementia knowledge and social comfort. Scores can range from 20 to 140, with a higher score indicating a more positive response. Compared with comparable instruments, this scale was found to possess good psychometric properties, including good reliability and convergent validity [11,22,28,30].

To enhance the validity of the ADKS and DAS, five content experts with experience in dementia and AD and health education were enlisted to review the suitability of the instruments for the present study. These experts were from Jordan, Australia, and Saudi Arabia; they possessed substantial expertise in AD and assessed the content validity of the instruments. In response to their feedback, no items were changed in either instrument. In addition, Cronbach’s alpha coefficient scores demonstrate that the internal consistency of the ADKS and DAS were 0.80 and 0.82, respectively, which confirms that these instruments are reliable. The questionnaire was distributed in its original English-language format.

Data analysis

Descriptive and inferential statistics were used to assess the sociodemographic and education-related characteristics of the respondents. Means and standard deviations were calculated to identify attitudes toward dementia of respondents. Both the Kolmogorov-Smirnov (p < 0.001) and Shapiro-Wilk (p < 0.001) tests were significant. Therefore, non-parametric tests were used, such as the Mann-Whitney test and the Kruskal-Wallis test. All statistical analyses were performed at a 0.05 level of significance.

## Results

Participants’ characteristics

Participants’ demographic characteristics conveyed that most were female (55.8%), and about 60.4% were between the ages of 20 and less than 25 years. Regarding the academic level, over half of the participants (53% of the students) were in their fourth year, while the rest were in their third year of study. The respondents in this study represented our universities: Taif University (28.7%), Majmaah University (28.5%), Al-Jouf University (22.2%), and Hafer Al-Batin University (20.5%). Only 14% of the respondents had a family history of Alzheimer's, and about 19.7% of the respondents had educated patients who had Alzheimer’s. In addition, about 29.6% of respondents reported having experience caring for people with Alzheimer’s, while 22.2% of the participants had experience caring for people with AD during clinical training (see Table 1).

**Table 1:** Mean scores and SD for content domains ADKS: Alzheimer’s Disease Knowledge Scale

Content domain	Minimum score	Maximum score	Mean score	SD
Life impact (score out of 3)	00	3.00	1.63 out of 3	0.91
Risk factors (score out of 6)	00	6.00	2.59 out of 6	1.13
Symptoms (score out of 4)	00	4.00	1.82 out of 4	1.06
Treatment and management (score out of 4)	00	4.00	2.13 out of 4	0.93
Assessment and diagnosis (score out of 4)	00	4.00	1.95 out of 4	0.92
Caregiving (score out of 5)	00	5.00	2.06 out of 5	1.11
Course of the disease (score out of 4)	00	4.00	2.13 out of 4	0.88
Total ADKS (score out of 30)	4.00	22.00	13.83 out of 30	3.29

Saudi Arabian nursing students’ knowledge of AD

According to Table 2, the mean score of the total ADKS sample is 13.83 out of a possible 30 (46%), which signifies that nursing students in this study had inadequate knowledge of AD. No nursing student reported having knowledge of all the knowledge items. The greatest number of correct answers were for question 12, poor nutrition can make the symptoms of AD worse; question 17, eventually, a person with AD will eventually need 24-hour supervision; and question 14, a person with AD becomes increasingly likely to fall down as the disease gets worse, with percentages of correct answers of 78.2%, 74.2%, and 73%, respectively. Conversely, the lowest numbers of correct scores were for question 6, when people with AD begin to have difficulty taking care of themselves, caregivers should take over right away; question 24, When a person has AD, using reminder notes is a crutch that can contribute to decline; and question 2, it has been scientifically proven that mental exercise can prevent a person from getting AD, with percentages of correct answers of 19.1%, 16.4%, and 7.1%, respectively. For more information, refer to Table 2 and supplementary tables in the Appendix.

**Table 2:** Mean scores and SD for content domains of the DAS DAS: Dementia Attitudes Scale

Content domain	Number of items	Minimum score	Maximum score	Mean score	SD
Social comfort (out of 70)	10	00	70	44.90	8.39
Dementia knowledge (out of 70)	10	00	70	54.39	9.82
Total attitude scale score (out of 140)	20	00	140	99.29	14.80

The knowledge of nursing students about the four domains of ADKS was insufficient. Inadequate knowledge about AD was exposed for the risk factors domain (a mean of 2.59 ± 1.13 out of 6), symptoms (a mean of 1.82 ± 1.06 out of 4), assessment and diagnosis (a mean of 1.95 ± 0.92 out of 4), and caregiving (a mean of 2.06 ± 1.11 out of 5). Regarding the other three domains, the students answered only slightly more than 50% of the questions correctly, which represents a low level of knowledge about AD. The mean score for the life impact domain is 1.63 ± 0.91 out of 3, for the treatment and management domain it is 2.13 ± 0.93 out of 4) and, for the course of disease domain, 2.13 ± 0.88 out of 4 (see Table 2).

Attitudes of Saudi Arabian nursing students toward AD

Table 3 and Supplementary Table 6 (in the Appendix) report the scores of respondents on the DAS. The overall mean DAS score is 99.29 out of 140 (71%); the mean score for the social comfort-related subdomain is 44.9 out of 70 (64.14%) and the subdomain for dementia knowledge has a score of 54.40 out of 70 (69%).

**Table 3:** Significant differences between mean total ADKS scores in relation to nursing students’ demographic characteristics for two groups, Mann-Whitney U test; for three or more groups, Kruskal-Wallis H statistics. AD: Alzheimer’s Disease; ADKS: Alzheimer’s Disease Knowledge Scale

Characteristics	Variables	Mean rank	Statistics	z/df	p
Gender	Male	233.49	26,900	-0.783	0.43
	Female	243.37			
Age group	Less than 20 years	251.32	2.855	2	0.24
	20 to less than 25 years	235.1			
	25 years and more	209.7			
Current academic year	Third year	254.35	24,897.0	‑2.304	0.021
	Fourth year	225.41			
University	Majmaah University	229.31	4.376	3	0.224
	Jouf University	240.57			
	Hafer Al-Batin University	263.32			
	Taif University	230.01			
Does anyone in your family have a history of Alzheimer’s?	yes	301.02	9,579.5	‑3.999	<0.001
	No	228.86			
Have you previously educated patients suffering from Alzheimer’s?	yes	260.77	15,954.5	‑1.720	0.085
	No	233.66			
Do you have experience caring with people with Alzheimer’s?	Yes	231.42	22,619.0	‑0.783	0.433
	No	242.18			
Do you have experience in caring for people with AD during clinical training?	yes	228.86	18,588.5	‑0.864	388
	No	241.9			

Correlation between mean total ADKS scores and nursing students’ demographic characteristics

Table 4 reveals significant disparities in the mean total score of the ADKS according to nursing students’ demographic characteristics. Nursing students in their third year had significantly higher ADKS scores than students in their fourth year of study (U = 24,897.000, p < 0.001), as indicated by a mean rank ADKS score of 254.35 for students in their third year and 225.41 for students in their fourth year. In addition, students with family members who had AD had a significantly higher ADKS score than students who did not have family members with this disease (U = 9,579.000, p = 0.021), with mean rank ADKS scores of 301.2 and 228.86, respectively.

**Table 4:** Significant differences between mean total DAS scores in relation to nursing students’ demographic characteristics for two groups, Mann-Whitney U test; for three or more groups, Kruskal-Wallis H statistics. DAS: Dementia Attitudes Scale; AD: Alzheimer’s Disease

Characteristics	Variables	Mean rank	Statistics	z/df	p
Gender	Male	218.32	23,700.0	‑2.919	0.004
	Female	255.4			
Age group	Less than 20 years	242.25	6.123	2	0.047
	20 to less than 25 years	243.37			
	25 years and more	177.67			
Current academic year	Third year	222.97	24,745.0	-2.391	0.017
	Fourth year	253.19			
University	Majmaah University	227.58	1.396	3	0.706
	Jouf University	240.79			
	Hafer Al-Batin University	242.99			
	Taif University	246.09			
Does anyone in your family have a history of Alzheimer’s?	yes	249.22	13,050.0	-0.655	0.512
	No	237.33			
Have you previously educated patients suffering from Alzheimer’s?	yes	247.56	17,196.0	-0.672	0.501
	No	236.9			
Do you have experience caring with people with Alzheimer’s?	Yes	252.56	21,775.5	-1.393	0.164
	No	233.31			
Do you have experience in caring for people with AD during clinical training?	yes	252.45	18,237.0	-1.140	0.254
	No	235.16			

Correlation between mean total DAS scores and nursing students’ demographic characteristics

Table 5 reveals marked variations in the mean total scores of the DAS. The results indicate a significantly higher DAS score for female participants than for male participants (U = 23,700.000, p = 0.004), with a mean rank DAS score of 255.40 for females and 218.32 for males. Furthermore, there is a statistically significant difference in the DAS scores for the three age groups of the students, χ^2^(^2^) = 6.123, p = 0.047. As illustrated in Table 5, the age group of 20 to 24 years had a higher mean rank DAS score of 243.37 than the other age groups. Finally, students in their fourth year had a significantly higher DAS score than third-year students (U = 24,745.000, p = 0.017), with a mean rank DAS score of 253.19 for students in their fourth year and 222.97 for students in their third year.

## Discussion

This study aimed to evaluate the knowledge and attitudes of undergraduate nursing students in Saudi Arabia toward people with AD. The findings of this study revealed that nursing students' knowledge of AD in Saudi Arabia was insufficient as indicated by their mean score of 13.83 out of 30 on the ADKS. All the ADKS subdomains reflect low scores for knowledge of risk factors, symptoms, assessment and diagnosis, caregiving, course of the disease, and treatment and management. These results are similar to those of studies undertaken in Nepal [31], Greece [17], England [18], India [12,19], and China [16,22] which also found nursing students’ knowledge about AD to be insufficient. Recently, Aljezawi et al. [32] reported similar results; this study of 275 Jordanian nursing students reports an average score on the ADKS of 18.3 out of 30, which indicates deficient knowledge regarding AD. The subdomains of the ADKS also yielded consistently low scores that ranged from 2.9 out of 4 for the subdomain course of disease, to 2.3 out of 5 for the caregiving subdomain. However, other studies, in Malta [11], South Korea [14], and Spain [13] found that nursing students have adequate knowledge of AD.

The current study reveals a notable deficiency in AD knowledge and care of nursing students. Students’ lack of knowledge can be attributed to gaps in the curricula of nursing schools in Saudi Arabia concerning AD and related content. Other studies confirm the deficiency of educational content on AD and ways to provide optimal nursing care for those affected by it in the curricula of nursing schools [13,33]. Therefore, updating and enhancing education and training are essential, to bolster knowledge regarding dementia-related illnesses [22,33]. The predominant teaching methods at Saudi universities are traditional lecture-based instruction, leading to the rote memorization of information for exam purposes. This approach may hinder the long-term retention and understanding of AD-related concepts.

In addition, the lack of practical exposure to caring for people with AD during the educational program could be a factor contributing to nursing students’ poor knowledge of AD, particularly after the COVID-19 pandemic; when the number of students in Saudi Arabia enrolling in nursing programs increased from 6,000 to over 19,000 [34]. This situation negatively affects the availability of training opportunities and adequate places where students can undergo clinical training. The importance of training nursing students to take care of AD patients is confirmed by Kimzey et al. [35], who report that nursing students who underwent clinical training with Alzheimer’s patients exhibited greater knowledge of and more positive attitudes toward AD patients than students who either completed an online module or received no training related to AD.

Furthermore, this study found that even nursing students who lacked adequate knowledge of AD had positive attitudes toward individuals with AD. This finding aligns with the literature, which also reports positive attitudes toward individuals with AD [11,12,17,19,21,22]. The positive attitudes found in nursing students in Saudi Arabia may be related to the Saudi population’s cultural values, particularly their reverence for older individuals, which is ingrained in Islamic and Arabic cultures. This is evident in one study conducted by Alshehry et al. [36] that examined the influence of Saudi nursing students' religiosity on their attitudes toward older people. The study reported that the students' views toward older persons and their judgments about senior care were positively influenced by religiosity [36]. In addition, this result may reflect students’ readiness and capability to care for individuals with AD. Notably, despite this positive attitude, nursing students lacked knowledge of AD, as confirmed by previous findings. One study reports that nurses had a negative attitude about working with AD patients [23].

The present study found a significant difference in AD knowledge among nursing students based on their academic levels. Surprisingly, in this study, third-year students exhibited slightly more AD knowledge than fourth-year students, in contrast to other studies [11-13]. A possible explanation for this result may be related to the timing and content of the nursing curriculum. At most universities in Saudi Arabia, the third-year curriculum tends to offer a more comprehensive approach to AD education, especially in medical-surgical modules. Hence, students in their third year are likely to have more knowledge of AD.

This study found that students with family members who had AD had significantly higher ADKS scores than students who did not, which may be attributed to first-hand experiences and personal connections, which foster a deeper understanding of the disease. This result confirms the findings of other studies [11,37], which found that nursing students who had previously been exposed to patients with dementia and related diseases reported feeling more capable of handling the associated challenges and demonstrated little apprehension about working with dementia patients, which led them to have better knowledge of AD. Hence, these students were more comfortable and prepared to work independently, with enhanced competency and empathy, with dementia patients during their careers in the future [37].

This study reports significantly different DAS scores for nursing students differentiated according to gender, age, and current academic year. First, females exhibited significantly higher DAS scores than males, which indicates a more positive attitude toward dementia among women, which corroborates the literature [11]. Second, there was a statistically significant difference in DAS scores for participants of different age groups, with those aged 20 to less than 25 years scoring higher than the other age groups [9,20]. Finally, students in their fourth years depicted notably higher DAS scores than those in their third year. This result is consistent with other studies [11,22], possibly because, by their fourth year of training, nursing students had had more exposure to AD, which caused them to have more positive attitudes.

Although the current study produced valuable results regarding knowledge of and attitudes toward AD among nursing students in Saudi Arabia, the results are subject to certain limitations. First, using a descriptive cross-sectional design does not allow causal relationships between variables to be established. Second, relying solely on self-reported questionnaires to assess nursing students’ knowledge of and attitudes toward AD may overlook important nuances. Incorporating other research methods, such as qualitative or observational methods, in a similar study could provide a more comprehensive understanding. Third, involving a convenience sample of nursing students in this study restricts the generalizability of the findings to other populations in Saudi Arabia.

## Conclusions

This study highlights the inadequacy of AD knowledge among nursing students in Saudi Arabia and emphasizes the need for interventions to enhance students’ understanding and management of this disease. In addition, this study found that students had positive attitudes toward caring for and working with AD patients. These findings underscore the importance of addressing this knowledge gap through targeted strategies in nursing practice, policy, and education. There is a need to develop and implement educational initiatives that are tailored to improving the AD knowledge of undergraduate nursing students. This involves ensuring that nursing schools offer high-quality AD education in their curricula by incorporating relevant and up-to-date information. Furthermore, nurse educators should consider integrating frequent, evidence-based, and accessible AD education programs that address students’ specific needs and learning objectives.

Collaboration between nursing schools and hospitals can provide valuable opportunities for students to access established AD education programs and enhance their practical skills and knowledge. More training in hospitals, particularly with older patients who have dementia and related diseases, is an essential activity that nursing schools in Saudi Arabia must consider because it is expected to increase students’ knowledge about AD, which will improve their attitudes toward working with AD patients. Moreover, good supervision during hospital training is vital for students learning to provide care for Alzheimer’s patients. This supervision offers essential guidance and support and helps students comprehend the intricate aspects of Alzheimer’s care, including the specialized needs and hurdles faced by patients with the disease. Supervision facilitates immediate feedback and guidance and enables students to cultivate the necessary skills and attitudes for helping Alzheimer’s patients. It also ensures that students adhere to best practices in dementia care and promotes patient safety and well-being. Finally, continuous hospital education sessions are also recommended to strengthen nursing students’ competence regarding AD care.

## Appendices

**Table 5:** Attitude toward Alzheimer's disease and related dementias scale among nursing students in SA SA: Saudi Arabia

Items	Mean	SD
1. It is rewarding to work with people who have AD.	3.87	1.844
2. I am afraid of people with AD.	5.44	1.761
3. People with AD can be creative.	4.65	1.803
4. I feel confident around people with AD.	3.84	1.693
5. I am comfortable touching people with AD.	4.90	1.728
6. I feel uncomfortable being around people with AD.	5.08	1.781
7. Every person with AD has different needs.	5.77	1.432
8. I am not very familiar with AD.	3.47	1.804
9. I would avoid an agitated person with AD.	5.42	1.683
10. People with AD like having familiar things nearby.	5.67	1.424
11. It is important to know the past history of people with AD.	5.90	1.399
12. It is possible to enjoy interacting with people with AD.	5.44	1.420
13. I feel relaxed around people with AD.	4.56	1.523
14. People with AD can enjoy life.	5.34	1.630
15. People with AD can feel when others are kind to them.	5.73	1.521
16. I feel frustrated because I do not know how to help people with AD.	2.99	1.759
17. I cannot imagine caring for someone with AD.	5.33	1.761
18. I admire the coping skills of people with AD.	5.06	1.477
19. We can do a lot now to improve the lives of people with AD.	5.90	1.391
20. Difficult behaviors may be a form of communication for people with AD.	4.92	1.685
Total attitude scale score (out of 140)	99.2	14.7
Comfort scored (out of 70)	44.8	8.3
Knowledge scoree (out of 70)	54.3	9.8

**Table 6:** Percentage of respondents who provided accurate answers for each item, ADKS questionnaire total score and scores for individual content domains. On the ADKs questionnaire (n = 477) ADKS: Alzheimer’s Disease Knowledge Scale

ADKS item	Domain	Answer	Correct answer number (out of 477)	Correct answer percentage
1. People with Alzheimer’s disease are particularly prone to depression.	Life Impact	TRUE	255	53.5
2. It has been scientifically proven that mental exercise can prevent a person from getting Alzheimer’s disease.	Risk Factors	FALSE	91	19.1
3. After symptoms of Alzheimer’s disease appear, the average life expectancy is 6 to 12 years.	Course of the Disease	TRUE	139	29.1
4. When a person with Alzheimer’s disease becomes agitated, a medical examination might reveal other health problems that caused the agitation.	Assessment and Diagnosis	TRUE	233	48.8
5. People with Alzheimer’s disease do best with simple, instructions giving one step at a time.	Caregiving	TRUE	291	61
6. When people with Alzheimer’s disease begin to have difficulty taking care of themselves, caregivers should take over right away.	Caregiving	FALSE	34	7.1
7. If a person with Alzheimer’s disease becomes alert and agitated at night, a good strategy is to try to make sure that the person gets plenty of physical activity during the day	Caregiving	TRUE	259	54.3
8. In rare cases, people have recovered from Alzheimer’s disease.	Course of the Disease	FALSE	174	36.5
9. People whose Alzheimer’s disease is not yet severe can benefit from psychotherapy for depression and anxiety	Treatment and Management	TRUE	279	58.5
10. If trouble with memory and confused thinking appears suddenly, it is likely due to Alzheimer’s disease.	Assessment and Diagnosis	FALSE	198	
11. Most people with Alzheimer’s disease live in nursing homes.	Life Impact	FALSE	227	47.6
12. Poor nutrition can make the symptoms of Alzheimer’s disease worse.	Treatment and Management	True	373	47.6
13. People in their 30s can have Alzheimer’s disease.	Risk Factors	TRUE	227	
14. A person with Alzheimer’s disease becomes increasingly likely to fall down as the disease gets worse.	Course of the Disease	TRUE	348	73
15. When people with Alzheimer’s disease repeat the same question or story several times, it is helpful to remind them that they are repeating themselves.	Caregiving	FALSE	270	56.6
16. Once people have Alzheimer’s disease, they are no longer capable of making informed decisions about their own care.	Caregiving	FALSE	129	27
17. Eventually, a person with Alzheimer’s disease will need 24 hour supervision	Course of the Disease	TRUE	354	74.2
18. Having high cholesterol may increase a person’s risk of developing Alzheimer’s disease.	Risk Factors	TRUE	167	35
19. Tremor or shaking of the hands or arms is a common symptom in people with Alzheimer’s disease.	Symptoms	FALSE	190	39.8
20. Symptoms of severe depression can be mistaken for symptoms of Alzheimer’s disease.	Assessment and Diagnosis	TRUE	194	40.7
21. Alzheimer’s disease is one type of dementia	Assessment and Diagnosis	TRUE	303	63.5
22. Trouble handling money or paying bills is a common early symptom of Alzheimer’s disease.	Symptoms	TRUE	169	35.4
23. One symptom that can occur with Alzheimer’s disease is believing that other people are stealing one’s things	Symptoms	TRUE	230	48.2
24. When a person has Alzheimer’s disease, using reminder notes is a crutch that can contribute to decline.	Treatment and Management	FALSE	78	16.4
25. Prescription drugs that prevent Alzheimer’s disease are available	Risk Factors	FALSE	260	54.5
26. Having high blood pressure may increase a person’s risk of developing of Alzheimer’s disease.	Risk Factors	TRUE	246	51.6
27. Genes can only partially account for the development of Alzheimer’s disease.	Risk Factors	TRUE	243	50.9
28. It is safe for people with Alzheimer’s disease to drive, as long as they have a companion in the car at all times.	Life Impacts	FALSE	294	61.6
29. Alzheimer’s disease cannot be cured.	Treatment and Management	TRUE	288	60.4
30. Most people with Alzheimer’s disease remember recent events better than things that happened in the past.	Symptoms	FALSE	281	58.9
